# Supplementary material for: Chromatin mapping and single-cell immune profiling define the temporal dynamics of ibrutinib response in CLL
Source: Nat Commun. 2020 Jan 29;11:577. doi: 10.1038/s41467-019-14081-6 (PMC6989523; doi:10.1038/s41467-019-14081-6)
Supplement: Supplementary file 1 — Supplementary Information [file 41467_2019_14081_MOESM1_ESM.pdf]

Chromatin mapping and single-cell immune profiling define the temporal dynamics of ibrutinib response in CLL

André F. Rendeiro[1*], Thomas Krausgruber[1*], Nikolaus Fortelny[1], Fangwen Zhao[1,2], Thomas Penz[1], Matthias Farlik[1], Linda C. Schuster[1], Amelie Nemc[1], Szabolcs Tasnády[3], Marienn Réti[3], Zoltán Mátrai[3], Donat Alpar[1,4†], Csaba Bödör[4†], Christian Schmidl[1,7†], Christoph Bock[1,2,5,6†]

[1] CeMM Research Center for Molecular Medicine of the Austrian Academy of Sciences, Vienna, Austria
[2] Ludwig Boltzmann Institute for Rare and Undiagnosed Diseases, Vienna, Austria
[3] Department of Haematology and Stem Cell Transplantation, Central Hospital of Southern Pest, National Institute of Hematology and Infectious Diseases, Budapest, Hungary
[4] MTA-SE Lendület Molecular Oncohematology Research Group, 1st Department of Pathology and Experimental Cancer Research, Semmelweis University, Budapest, Hungary
[5] Department of Laboratory Medicine, Medical University of Vienna, Vienna, Austria
[6] Max Planck Institute for Informatics, Saarland Informatics Campus, Saarbrücken, Germany
[7] Current address: Regensburg Center for Interventional Immunology (RCI), Regensburg, Germany

[*] These authors contributed equally to this work
[†] Co-last author / These authors jointly directed this work
Correspondence: Christoph Bock (cbock@cemm.oeaw.ac.at)

Keywords: Chronic lymphocytic leukemia, drug response profiling, ibrutinib therapy, chromatin mapping, single-cell RNA sequencing, time series analysis, machine learning, translational bioinformatics

Supplementary Figures

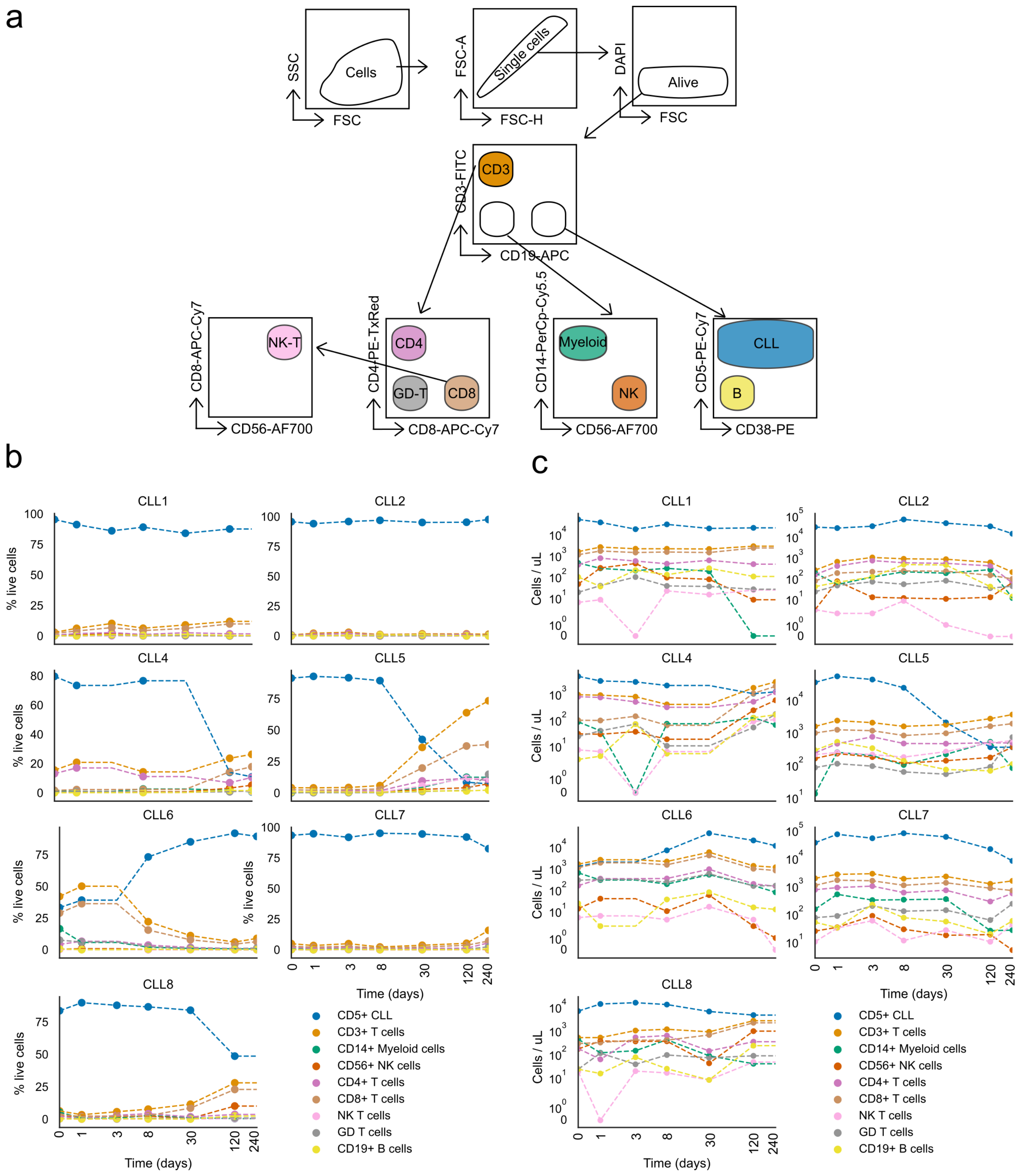

Supplementary Figure 1: Time series of cell composition in patients with CLL upon ibrutinib treatment

a) Schematic representation of the FACS approach for B cells (CD19+CD5-CD38-), CD4+ T cells (CD3+CD4+), CD8+ T cells (CD3+CD8+), CLL cells (CD19+CD5+), gamma-delta T-cells (CD3+CD4-CD8-), myeloid cells (CD3-CD19-

CD14+), NK cells (CD3-CD19-CD56+), NK-T cells (CD3+CD8+CD56+), and T cells (CD3+) from PBMCs of patients with CLL. This gating strategy was used to: (i) analyze the composition of the immune cell compartment (Fig. 1b-c); (ii) quantify surface marker expression (Fig. 1d); (iii) enrich cell populations for single-cell transcriptome analysis (Fig. 1e-i, Fig. 2d-f, Fig. 3e-f); and (iv) sort cell populations for bulk chromatin mapping (Fig. 2a-e, Fig. 3a-d). **b-c)** Flow cytometry quantification of the relative (b) or absolute (c) abundance of CLL cells and several non-malignant immune cell types in patients undergoing ibrutinib therapy. In total, 45 samples obtained from seven patients were included in the analysis.

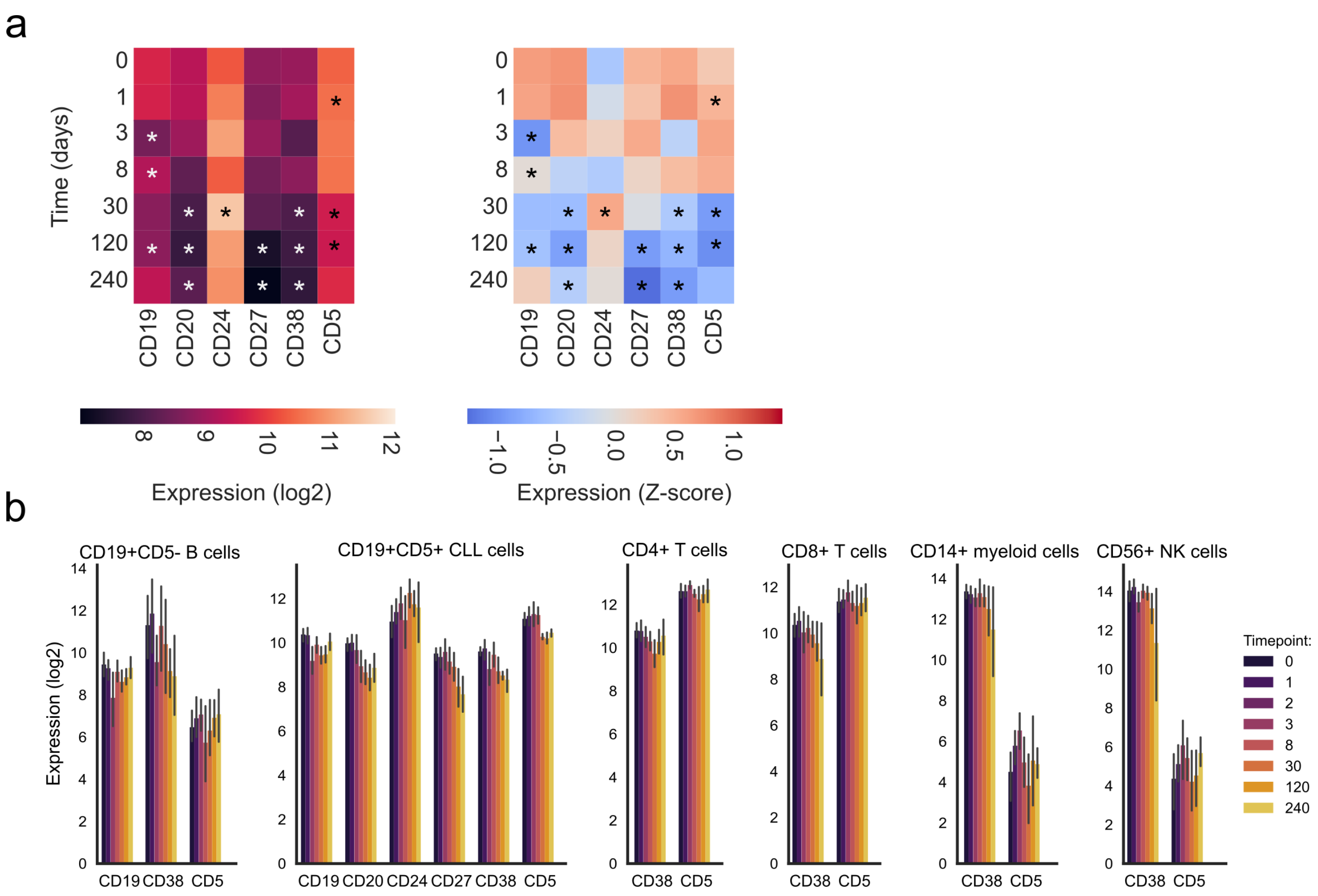

Supplementary Figure 2: Temporal dynamics of T cell subsets and surface marker expression

a) Mean expression of surface marker proteins in CD19^{+}CD5^{+} CLL cells during ibrutinib treatment. The left panel displays absolute (log scale) expression, while the right panel displays column-wise Z-transformed values. Stars mark significant changes compared to time 0 (paired t-test, $p < 0.05$). **b)** Expression of surface marker proteins in immune cell subsets of patients with CLL as measured by flow cytometry (error bars represent 95 percent confidence intervals of the mean). In total, 45 samples obtained from seven patients were included in the analysis.

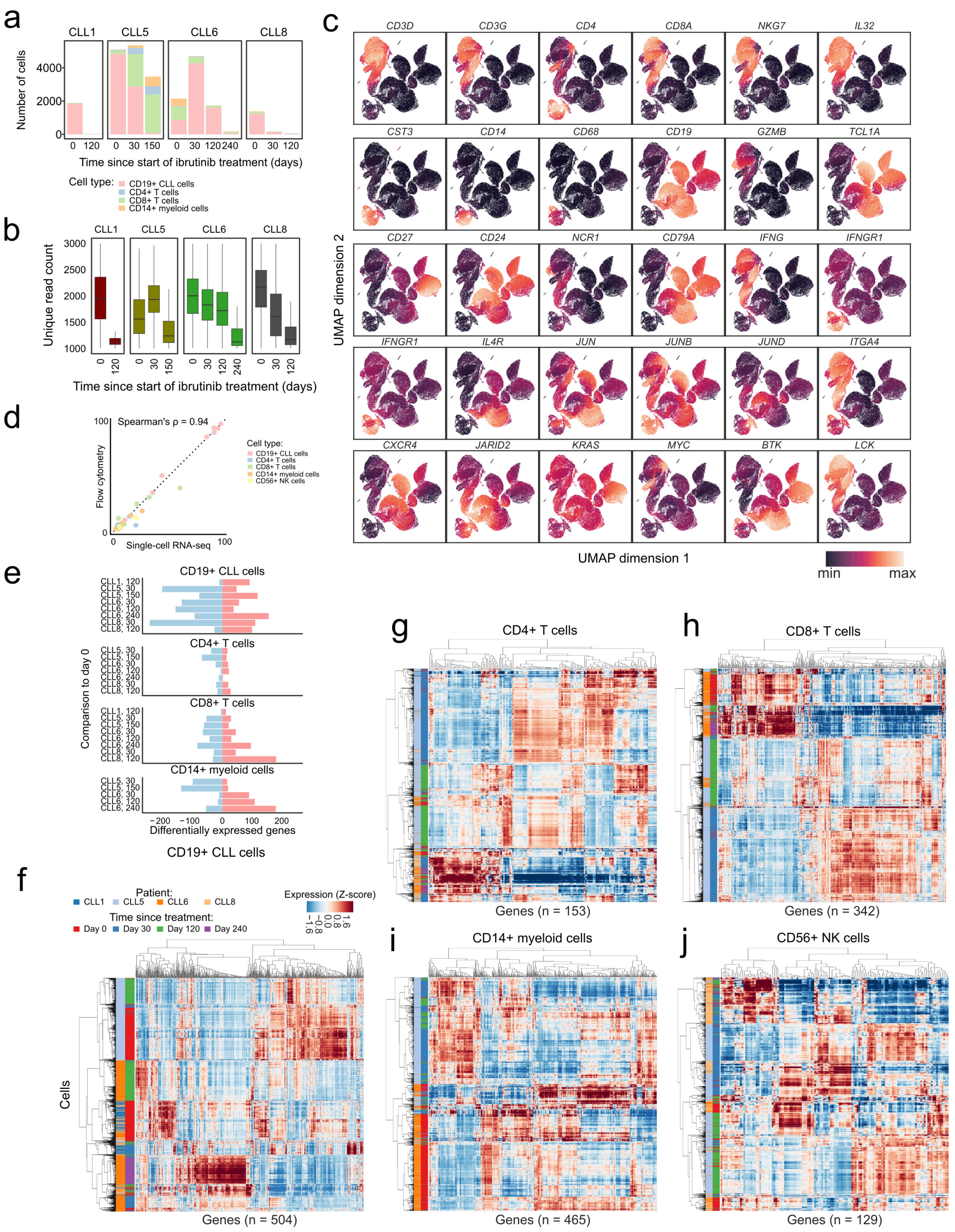
a
CLL1
CLL5
CLL6
CLL8
Number of cells
Time since start of ibrutinib treatment (days)
Cell type:
CD19+ CLL cells
CD4+ T cells
CD8+ T cells
CD14+ myeloid cells
b
Unique read count
Time since start of ibrutinib treatment (days)
c
CD3D CD3G CD4 CD8A NKG7 IL32
CST3 CD14 CD68 CD19 GZMB TCL1A
CD27 CD24 NCR1 CD79A IFNG IFNGR1
IFNGR1 IL4R JUN JUNB JUND ITGA4
CXCR4 JARID2 KRAS MYC BTK LCK
UMAP dimension 2
UMAP dimension 1
min
max
d
Spearman's ρ = 0.94
Flow cytometry
Single-cell RNA-seq
Cell type:
CD19+ CLL cells
CD4+ T cells
CD8+ T cells
CD14+ myeloid cells
CD56+ NK cells
e
CD19+ CLL cells
CD4+ T cells
CD8+ T cells
CD14+ myeloid cells
Comparison to day 0
Differentially expressed genes
f
CD19+ CLL cells
Patient:
CLL1 CLL5 CLL6 CLL8
Time since treatment:
Day 0 Day 30 Day 120 Day 240
Expression (Z-score)
Cells
Genes (n = 504)
g
CD4+ T cells
Genes (n = 153)
h
CD8+ T cells
Genes (n = 342)
i
CD14+ myeloid cells
Genes (n = 465)
j
CD56+ NK cells
Genes (n = 129)

Supplementary Figure 3: Single-cell RNA-seq profiling over the ibrutinib time course

a) Bar plot displaying the number of single-cell transcriptome profiles that passed quality control, shown separately for each patient, cell type, and time point. **b)** Box plot displaying the number of unique molecular identifiers (UMIs) detected per single cell, shown separately for each patient, cell type, and time point. **c)** Heatmap showing mean expression levels of the marker genes that were used to assign the single-cell transcriptomes to defined cell types. Values represent expression levels (normalized UMI counts) scaled from minimum to maximum in each row. **d**) Scatterplot comparing the fraction of cells of each type based on single-cell RNA-seq versus flow cytometry across all patients and time points. **e)** Number of differentially expressed genes for each cell type, patient, and time point. **f-j)** Heatmaps of differentially expressed genes over the ibrutinib time course separately for each cell type.

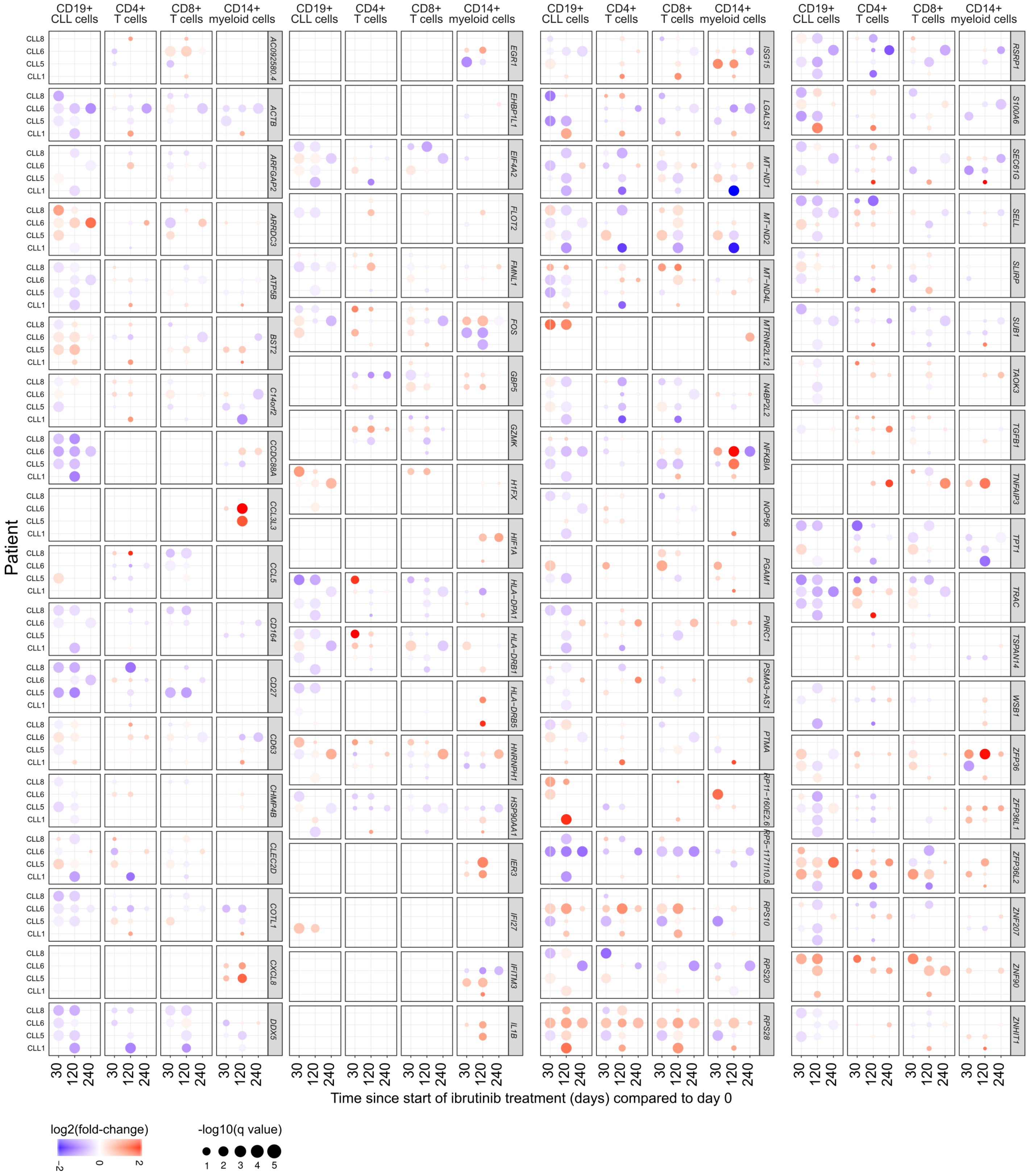

Supplementary Figure 4: Transcriptional changes of differentially expressed genes upon ibrutinib treatment

Differential gene expression for those genes that were significantly differentially expressed (absolute log fold change higher than one in more than two patients) over the course of ibrutinib therapy, shown separately for each cell type and comparing each sample to the pre-treatment (day 0) sample from the same patient.

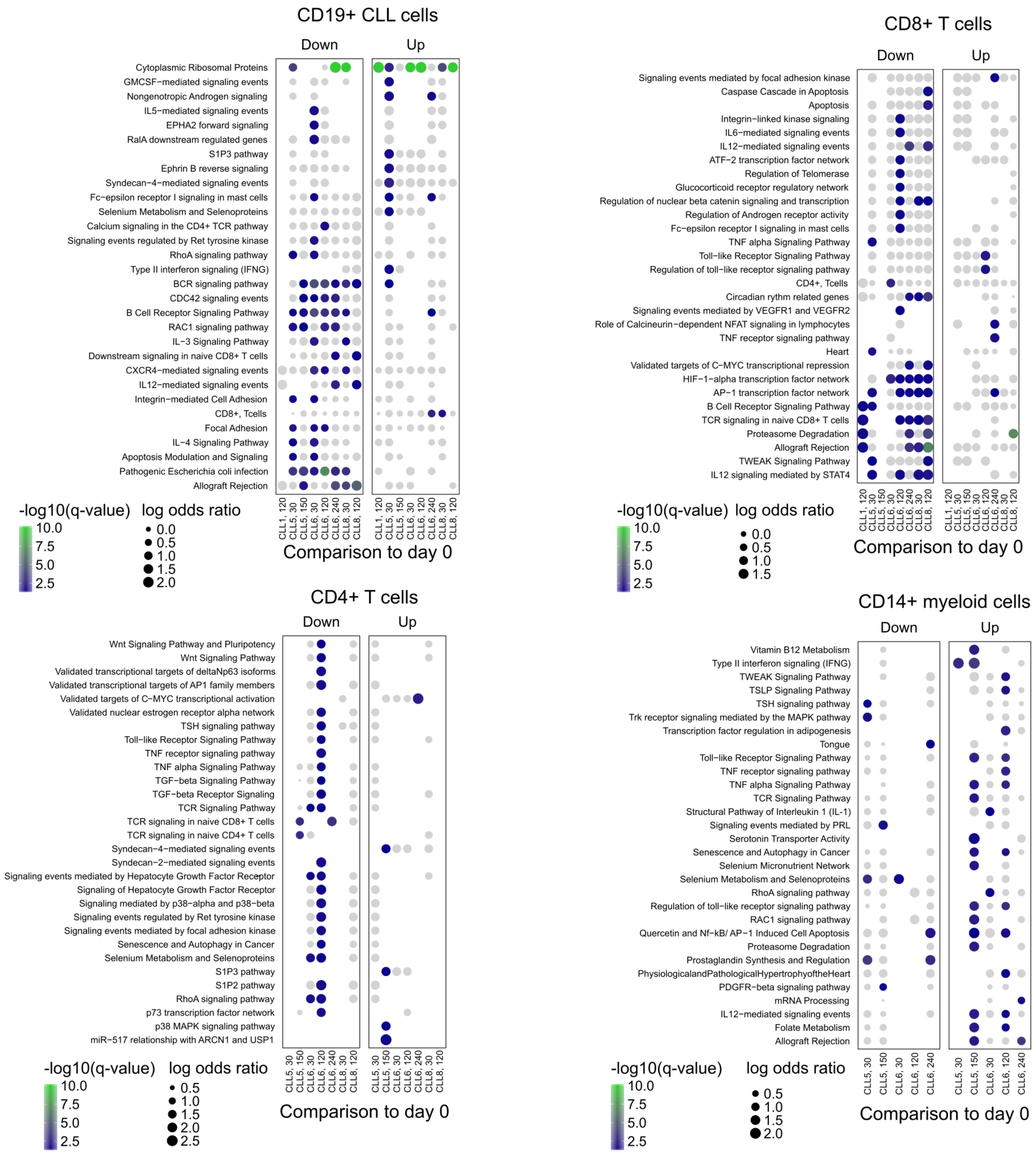

Supplementary Figure 5: Gene set enrichments for single-cell RNA-seq over the ibrutinib time course

Enrichment of differentially expressed genes over the course of ibrutinib therapy for gene sets and biological processes involved in immune regulation, calculated separately for each cell type.

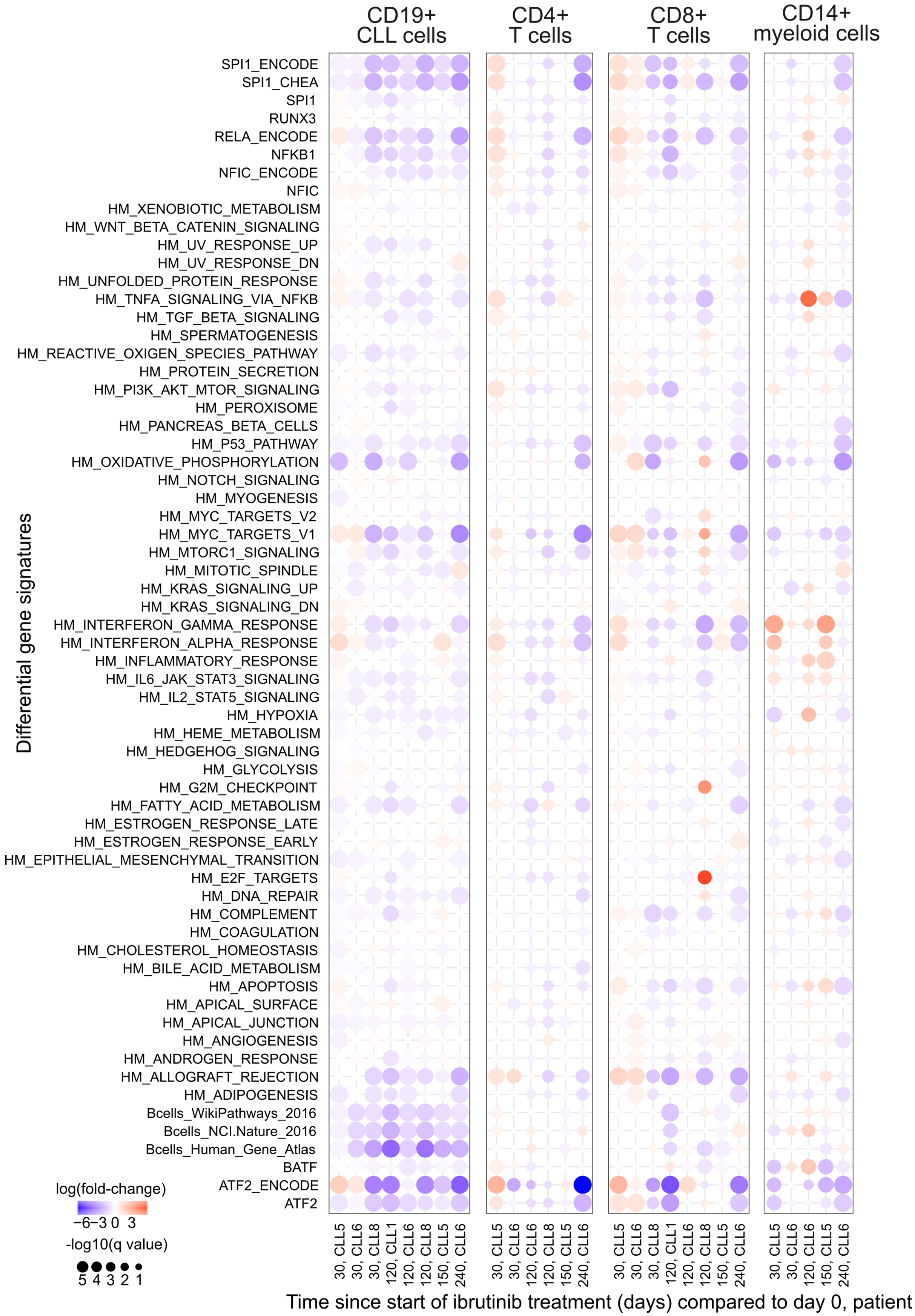

Supplementary Figure 6: Transcriptional changes of selected gene signatures upon ibrutinib treatment

Aggregate gene expression of selected gene signatures plotted over the ibrutinib time course. The list of gene signatures includes the hallmark signatures from MSigDB[59] (indicated by HM prefix) as well as sets of target genes for selected transcription factors (obtained from various sources).

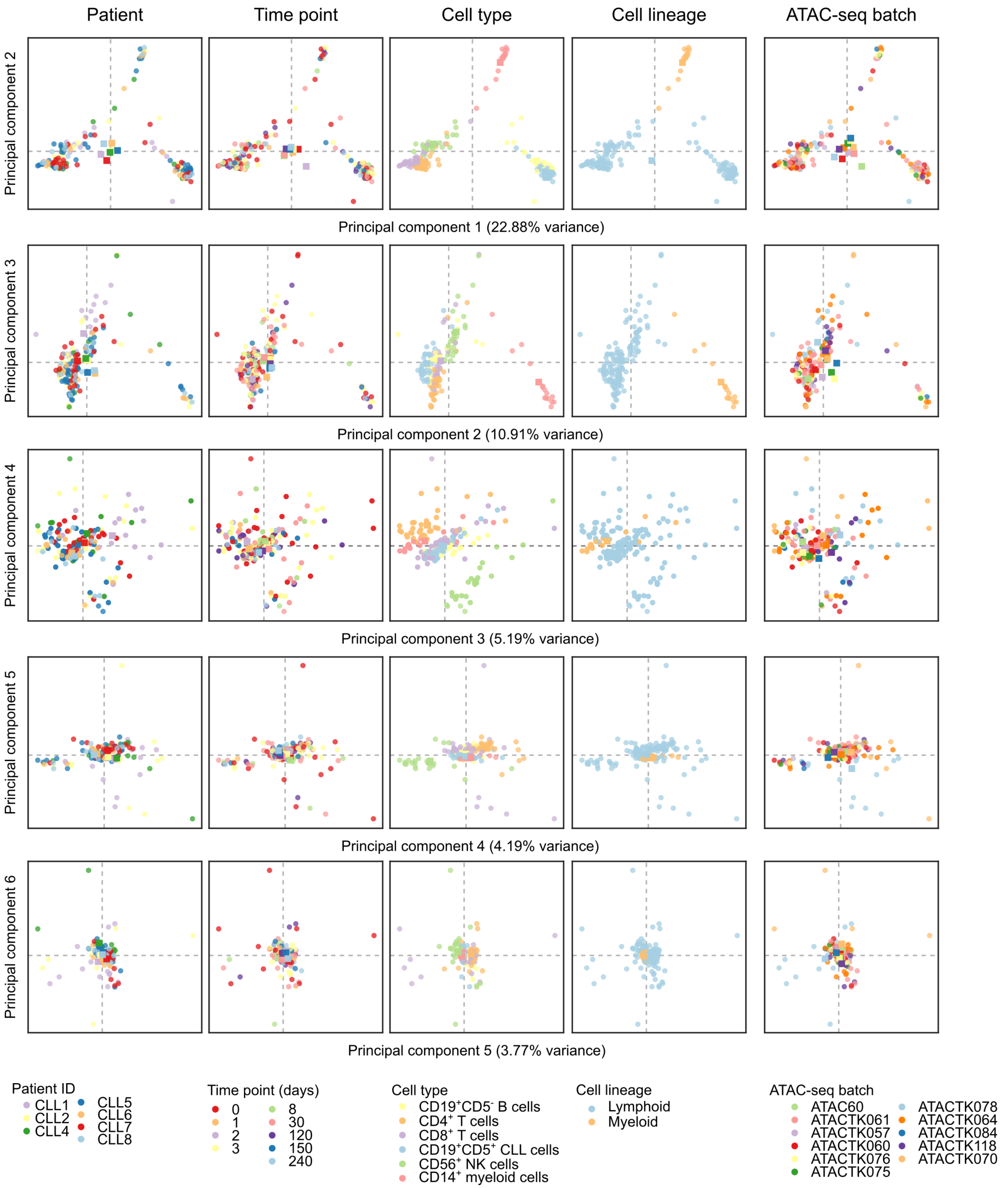

Supplementary Figure 7: Unsupervised analysis of chromatin accessibility over the ibrutinib time course

Principal component analysis of all chromatin accessibility profiles, highlighting biological and technical annotations of potential relevance. Samples are shown as circles, color-coded according to annotations, and the centroid for each annotation is shown as a color-coded square. In total, 155 samples obtained from seven patients were included in the analysis.

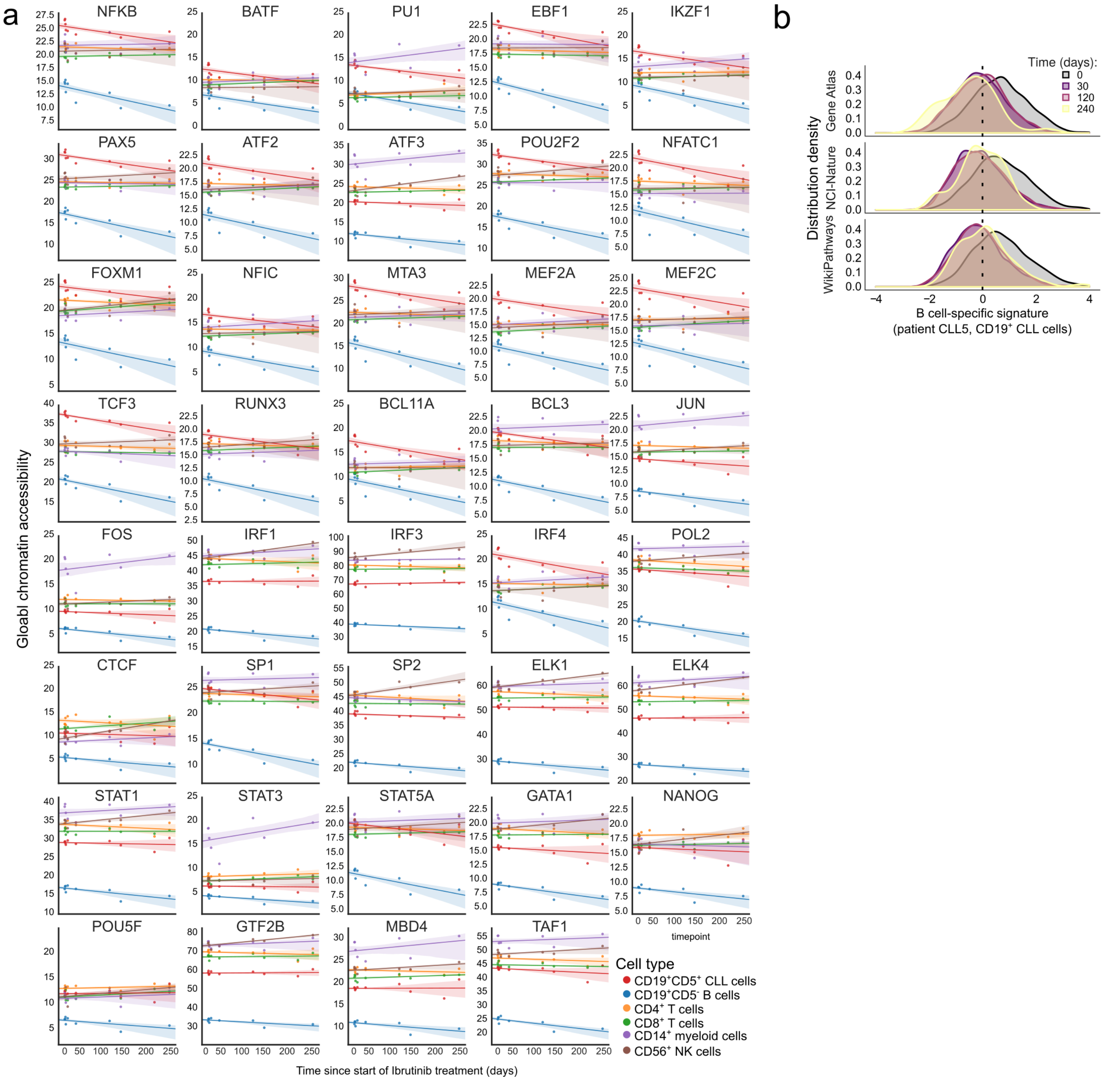

Supplementary Figure 8: Changes in transcription regulation and cell state over the ibrutinib time course

a) Line plots showing mean chromatin accessibility of regulatory regions overlapping putative binding sites of the respective transcription factors (based on publicly available ChIP-seq data) for each cell type and time point. Colored areas indicate 95 percent confidence intervals calculated over 1,000 bootstrap runs. 155 samples obtained from seven patients were included in the analysis, comprising CLL cells as well as non-malignant immune cells. **b)** Gene expression histogram across CLL cells in one patient, demonstrating the decline of B cell-specific expression signature (three alternative signatures are shown) over the time course of ibrutinib treatment. For illustration, the patient with most time points in the single-cell RNA-seq analysis (CLL5) is displayed.

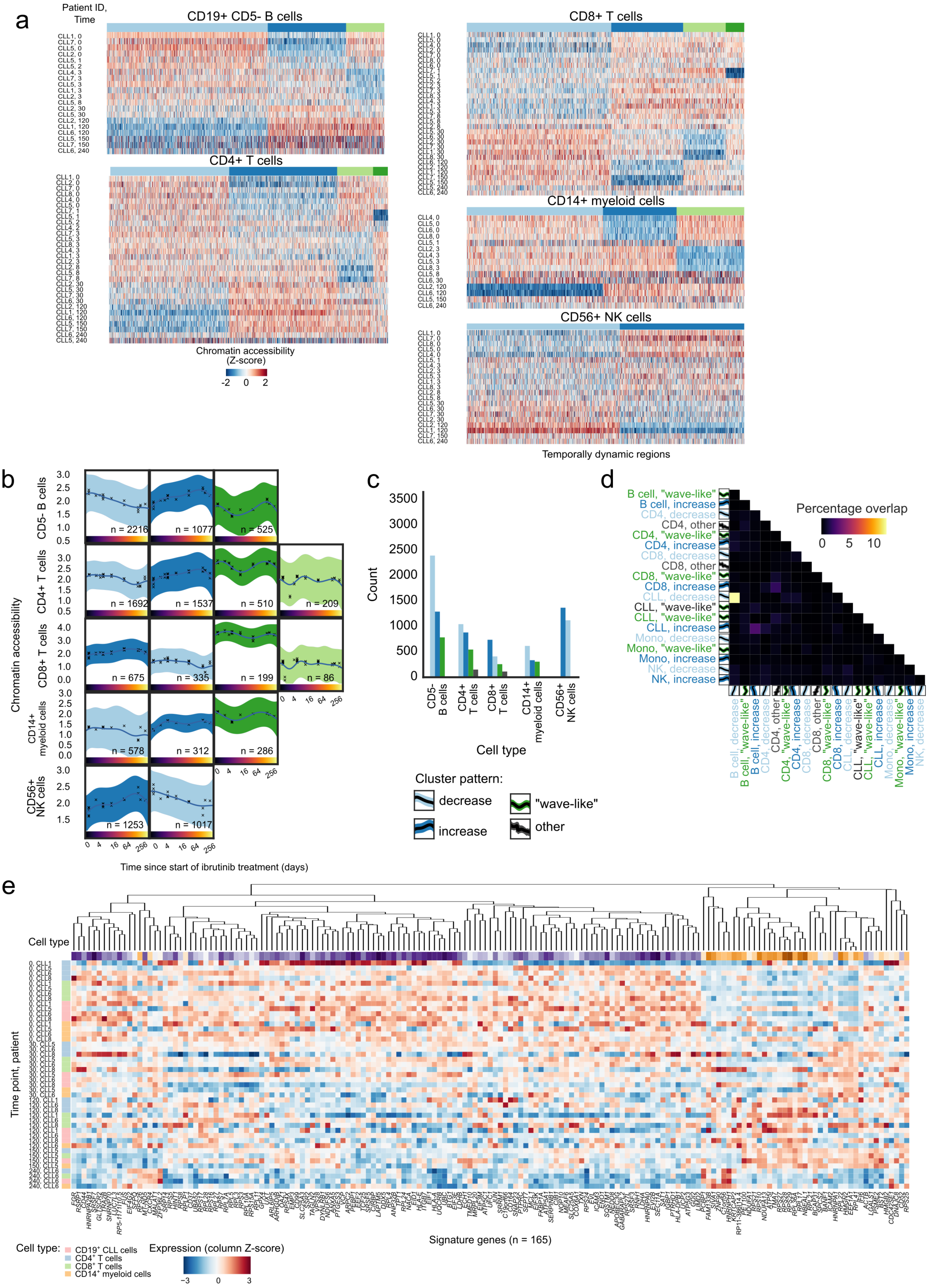
a
Patient ID, Time
CD19+ CD5- B cells
CD8+ T cells
CD4+ T cells
CD14+ myeloid cells
CD56+ NK cells
Chromatin accessibility (Z-score)
-2 0 2
Temporally dynamic regions
b
CD5- B cells
CD4+ T cells
CD8+ T cells
CD14+ myeloid cells
CD56+ NK cells
Chromatin accessibility
n = 2216
n = 1077
n = 525
n = 1692
n = 1537
n = 510
n = 209
n = 675
n = 335
n = 199
n = 86
n = 578
n = 312
n = 286
n = 1253
n = 1017
Time since start of ibrutinib treatment (days)
c
Count
CD5- B cells
CD4+ T cells
CD8+ T cells
CD14+ myeloid cells
CD56+ NK cells
Cell type
Cluster pattern:
decrease
"wave-like"
increase
other
d
Percentage overlap
0 5 10
B cell, "wave-like"
B cell, increase
CD4, decrease
CD4, other
CD4, "wave-like"
CD4, increase
CD8, decrease
CD8, other
CD8, "wave-like"
CD8, increase
CLL, decrease
CLL, "wave-like"
CLL, "wave-like"
CLL, increase
Mono, decrease
Mono, "wave-like"
Mono, increase
NK, decrease
NK, increase
e
Cell type
Time point, patient
Signature genes (n = 165)
Cell type:
CD19+ CLL cells
CD4+ T cells
CD8+ T cells
CD14+ myeloid cells
Expression (column Z-score)
−3 0 3

Supplementary Figure 9: Cluster analysis of regulatory regions in non-malignant immune cell types

a) Heatmaps showing chromatin accessibility at dynamically changing regulatory regions for five FACS-purified non-malignant immune cell types collected over the ibrutinib time course. Values represent column Z-scores of normalized ATAC-seq signal strength. 122 samples obtained from seven patients were included in the analysis, comprising only non-malignant immune cells. **b)** Mean chromatin accessibility across patients plotted over the ibrutinib time course for each cluster of dynamically changing regulatory regions in each cell type. Each cross represents a single sample from a single patient at a specific time point, and 95% confidence intervals are shown as colored shapes. **c)** Absolute number of dynamic regulatory regions for each cell type and cluster. **d)** Pairwise overlap of dynamic regulatory regions between cell types and clusters. **e)** Clustered heatmap showing patient-specific gene expression levels for the quiescence-like gene expression signature (**Figure 3e**), based on the single-cell RNA-seq data over the ibrutinib time course. Values represent column Z-scores of gene expression. 12 independent patient samples were included in the analysis.

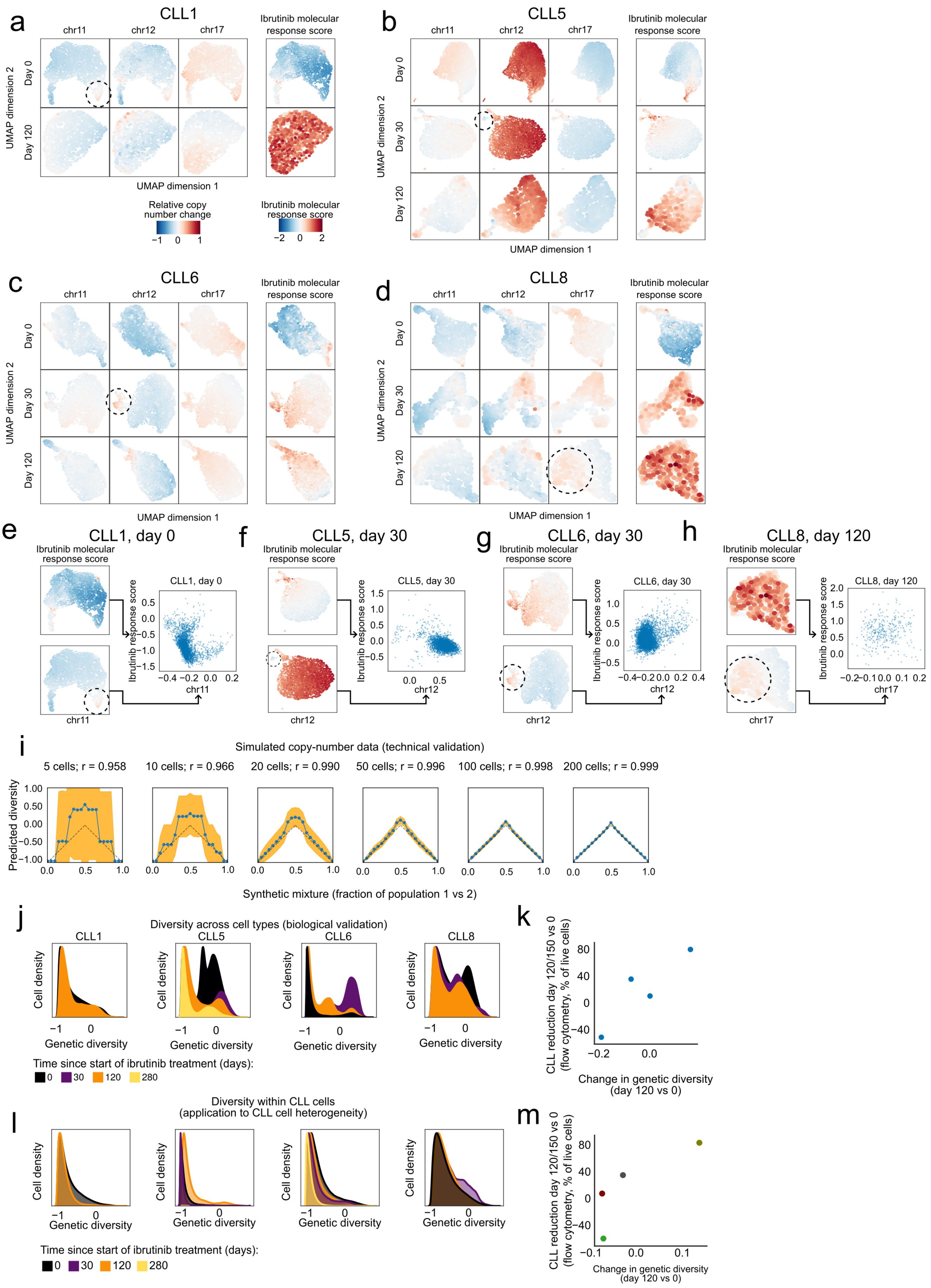
a
CLL1
chr11
chr12
chr17
Ibrutinib molecular response score
Day 0
Day 120
UMAP dimension 2
UMAP dimension 1
Relative copy number change
Ibrutinib molecular response score
b
CLL5
Day 30
c
CLL6
d
CLL8
e
CLL1, day 0
f
CLL5, day 30
g
CLL6, day 30
h
CLL8, day 120
Ibrutinib response score
i
Simulated copy-number data (technical validation)
5 cells; r = 0.958
10 cells; r = 0.966
20 cells; r = 0.990
50 cells; r = 0.996
100 cells; r = 0.998
200 cells; r = 0.999
Predicted diversity
Synthetic mixture (fraction of population 1 vs 2)
j
Diversity across cell types (biological validation)
Cell density
Genetic diversity
Time since start of ibrutinib treatment (days):
0
30
120
280
k
CLL reduction day 120/150 vs 0 (flow cytometry, % of live cells)
Change in genetic diversity (day 120 vs 0)
l
Diversity within CLL cells (application to CLL cell heterogeneity)
m

Supplementary Figure 10: Analysis of copy number and genetic diversity over the ibrutinib time course

a-d) Two-dimensional similarity map (UMAP projection) based on DNA copy number profiles for single cells inferred from the single-cell RNA-seq data. These maps were calculated separately for each patient and time point. Color-coding indicates the relative copy number change for three chromosomal aberrations common in CLL (left) and for the ibrutinib molecular response score (right), i.e., the change in the CLL cell percentage on day 120/150 of ibrutinib treatment compared to day 0 as measured by flow cytometry. Genetically distinct subclones are highlighted by dashed circles. **e-h)** Scatterplots comparing selected subclonal copy number aberrations (x-axis and UMAP plots on the bottom left) with the ibrutinib molecular response score (y-axis and UMAP plots on the top left) across single cells in individual patients and time points. **i)** Accuracy of the computational approach for quantifying genetic diversity benchmarked on simulated copy number profiles that were combined at defined percentages (x-axis). Dashed lines indicate expected values (based on the simulation's known ground truth), blue lines indicate inferred values, and yellow areas represents 95th confidence intervals for the inferred values. Correlation coefficients quantify the overall agreement between expected and inferred values. **j)** Histograms showing the change in genetic diversity across all cells (i.e., CLL cells and immune cells) in the single-cell RNA-seq dataset. **k**) Scatterplot showing the correlation between the change in genetic diversity across all cells (x-axis) between time points and the cellular response to ibrutinib treatment (y-axis). **l**) Histograms showing the change in genetic diversity specifically for CLL cells. **m**) Scatterplot showing the correlation between changes in genetic diversity specifically in CLL cells (x-axis) and the cellular response to ibrutinib treatment (y-axis). Panel m is a reproduction of Figure 4b for consistency with panels j-k.

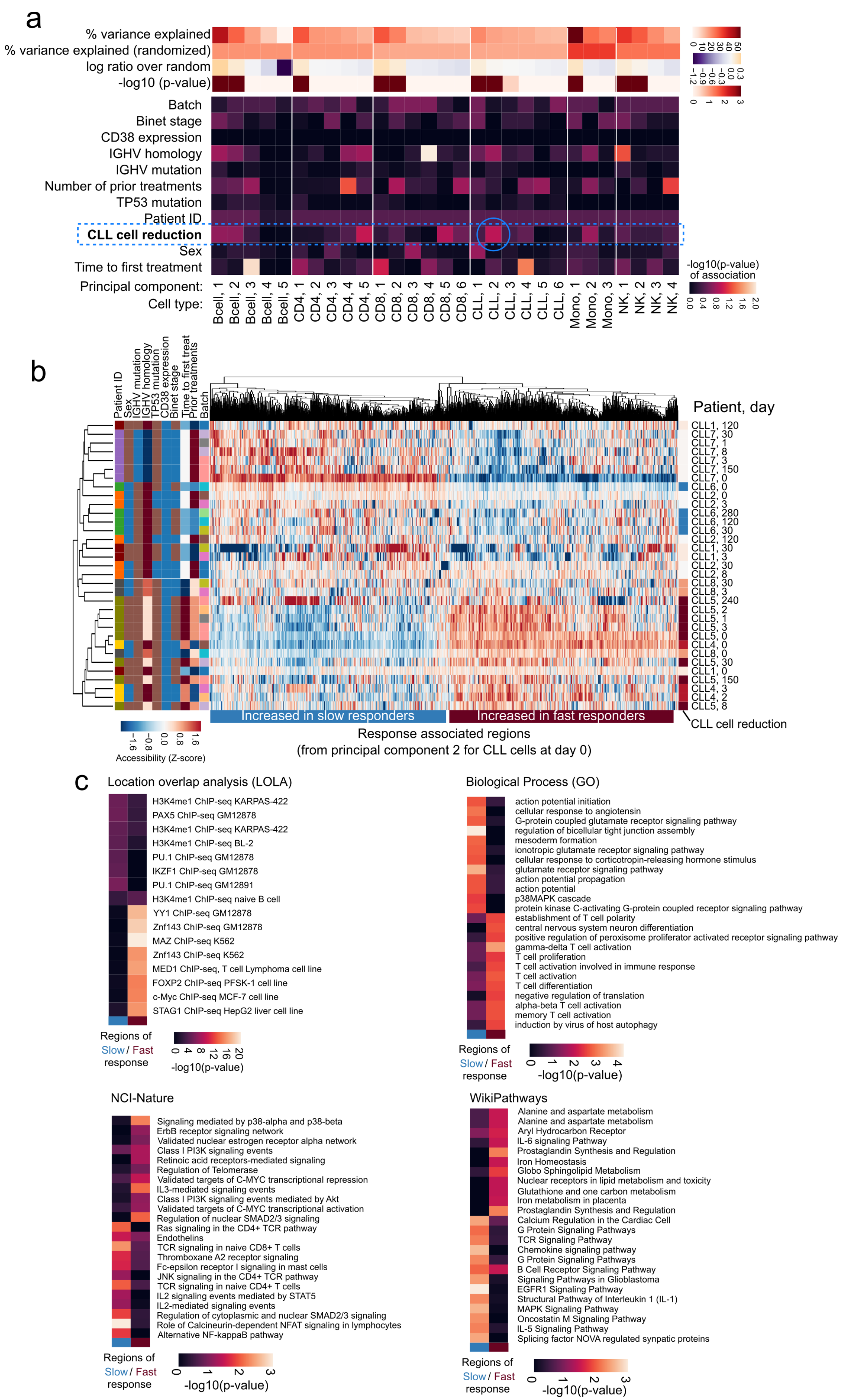
a
% variance explained
% variance explained (randomized)
log ratio over random
-log10 (p-value)
Batch
Binet stage
CD38 expression
IGHV homology
IGHV mutation
Number of prior treatments
TP53 mutation
Patient ID
CLL cell reduction
Sex
Time to first treatment
Principal component:
Cell type:
-log10(p-value) of association
b
Patient ID
Sex
IGHV mutation
IGHV homology
TP53 mutation
CD38 expression
Binet stage
Time to first treat
Prior treatments
Batch
Patient, day
CLL1, 120
CLL7, 30
CLL7, 1
CLL7, 8
CLL7, 3
CLL7, 150
CLL7, 0
CLL6, 0
CLL2, 0
CLL2, 3
CLL6, 280
CLL6, 120
CLL6, 30
CLL2, 120
CLL1, 30
CLL1, 3
CLL2, 30
CLL2, 8
CLL8, 30
CLL8, 3
CLL5, 240
CLL5, 2
CLL5, 1
CLL5, 3
CLL5, 0
CLL4, 0
CLL8, 0
CLL5, 30
CLL1, 0
CLL5, 150
CLL4, 3
CLL4, 2
CLL5, 8
Increased in slow responders
Increased in fast responders
CLL cell reduction
Accessibility (Z-score)
Response associated regions
(from principal component 2 for CLL cells at day 0)
c
Location overlap analysis (LOLA)
H3K4me1 ChIP-seq KARPAS-422
PAX5 ChIP-seq GM12878
H3K4me1 ChIP-seq KARPAS-422
H3K4me1 ChIP-seq BL-2
PU.1 ChIP-seq GM12878
IKZF1 ChIP-seq GM12878
PU.1 ChIP-seq GM12891
H3K4me1 ChIP-seq naive B cell
YY1 ChIP-seq GM12878
Znf143 ChIP-seq GM12878
MAZ ChIP-seq K562
Znf143 ChIP-seq K562
MED1 ChIP-seq, T cell Lymphoma cell line
FOXP2 ChIP-seq PFSK-1 cell line
c-Myc ChIP-seq MCF-7 cell line
STAG1 ChIP-seq HepG2 liver cell line
Regions of Slow / Fast response
-log10(p-value)
Biological Process (GO)
action potential initiation
cellular response to angiotensin
G-protein coupled glutamate receptor signaling pathway
regulation of bicellular tight junction assembly
mesoderm formation
ionotropic glutamate receptor signaling pathway
cellular response to corticotropin-releasing hormone stimulus
glutamate receptor signaling pathway
action potential propagation
action potential
p38MAPK cascade
protein kinase C-activating G-protein coupled receptor signaling pathway
establishment of T cell polarity
central nervous system neuron differentiation
positive regulation of peroxisome proliferator activated receptor signaling pathway
gamma-delta T cell activation
T cell proliferation
T cell activation involved in immune response
T cell activation
T cell differentiation
negative regulation of translation
alpha-beta T cell activation
memory T cell activation
induction by virus of host autophagy
Regions of Slow / Fast response
-log10(p-value)
NCI-Nature
Signaling mediated by p38-alpha and p38-beta
ErbB receptor signaling network
Validated nuclear estrogen receptor alpha network
Class I PI3K signaling events
Retinoic acid receptors-mediated signaling
Regulation of Telomerase
Validated targets of C-MYC transcriptional repression
IL3-mediated signaling events
Class I PI3K signaling events mediated by Akt
Validated targets of C-MYC transcriptional activation
Regulation of nuclear SMAD2/3 signaling
Ras signaling in the CD4+ TCR pathway
Endothelins
TCR signaling in naive CD8+ T cells
Thromboxane A2 receptor signaling
Fc-epsilon receptor I signaling in mast cells
JNK signaling in the CD4+ TCR pathway
TCR signaling in naive CD4+ T cells
IL2 signaling events mediated by STAT5
IL2-mediated signaling events
Regulation of cytoplasmic and nuclear SMAD2/3 signaling
Role of Calcineurin-dependent NFAT signaling in lymphocytes
Alternative NF-kappaB pathway
Regions of Slow / Fast response
-log10(p-value)
WikiPathways
Alanine and aspartate metabolism
Alanine and aspartate metabolism
Aryl Hydrocarbon Receptor
IL-6 signaling Pathway
Prostaglandin Synthesis and Regulation
Iron Homeostasis
Globo Sphingolipid Metabolism
Nuclear receptors in lipid metabolism and toxicity
Glutathione and one carbon metabolism
Iron metabolism in placenta
Prostaglandin Synthesis and Regulation
Calcium Regulation in the Cardiac Cell
G Protein Signaling Pathways
TCR Signaling Pathway
Chemokine signaling pathway
G Protein Signaling Pathways
B Cell Receptor Signaling Pathway
Signaling Pathways in Glioblastoma
EGFR1 Signaling Pathway
Structural Pathway of Interleukin 1 (IL-1)
MAPK Signaling Pathway
Oncostatin M Signaling Pathway
IL-5 Signaling Pathway
Splicing factor NOVA regulated synpatic proteins
Regions of Slow / Fast response
-log10(p-value)

Supplementary Figure 11: Analysis of chromatin profiles and their association with the response to ibrutinib

a) Heatmaps showing the association of various clinical annotations with the principal components of the cell type specific chromatin accessibility profiles of different cell types prior to the start of ibrutinib treatment. The blue circle highlights the association between the second principal component for CLL cells and the change in the CLL cell percentage on day 120/150 of ibrutinib treatment compared to day 0, as measured by flow cytometry (y-axis). 7 independent patient samples were included in the analysis. **b)** Clustered heatmap showing patient-specific chromatin profiles for genomic regions associated with the second principal component (from panel a). 33 samples obtained from seven patients were included in the analysis. **c)** Enrichment analysis for genomic regions associated with the second principal component, separately for regions associated with a slow versus a fast response to ibrutinib treatment.

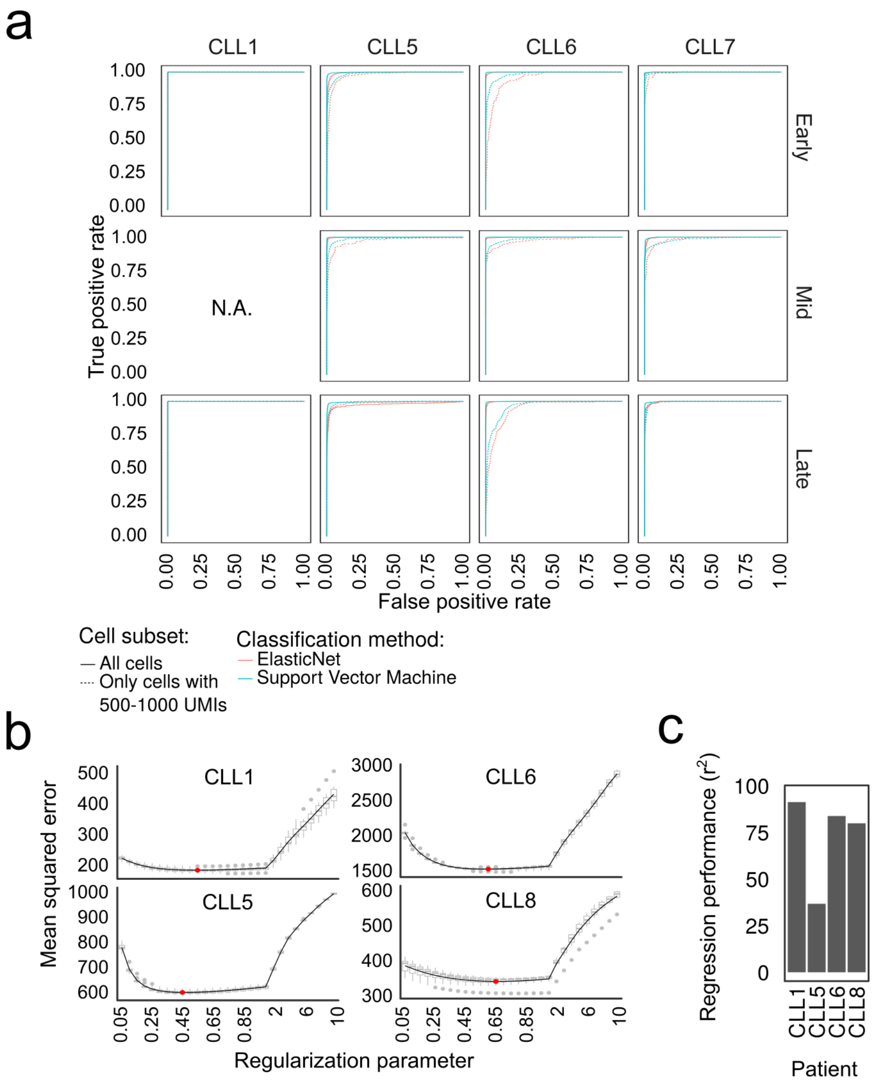

Supplementary Figure 12: Prediction of the time point of sample collection from single-cell transcriptomes

a) ROC curves showing the cross-validated test set performance of classifiers predicting the time point of sample collection based on single-cell transcriptome profiles, using two different machine learning methods (logistic regression with elastic net regularization and support vector machines) and two different thresholds for single-cell RNA-seq data quality (all cells vs. only cells with 500 to 1,000 UMIs). **b)** Optimization of the regularization parameter (lambda) for predicting the time since the start of ibrutinib treatment using elastic net regularized linear regression. Red dots indicate the chosen parameter for each patient. **c)** Cross-validated test set performance of the regression models (coefficient of determination) for predicting the time since the start of ibrutinib treatment for each patient.

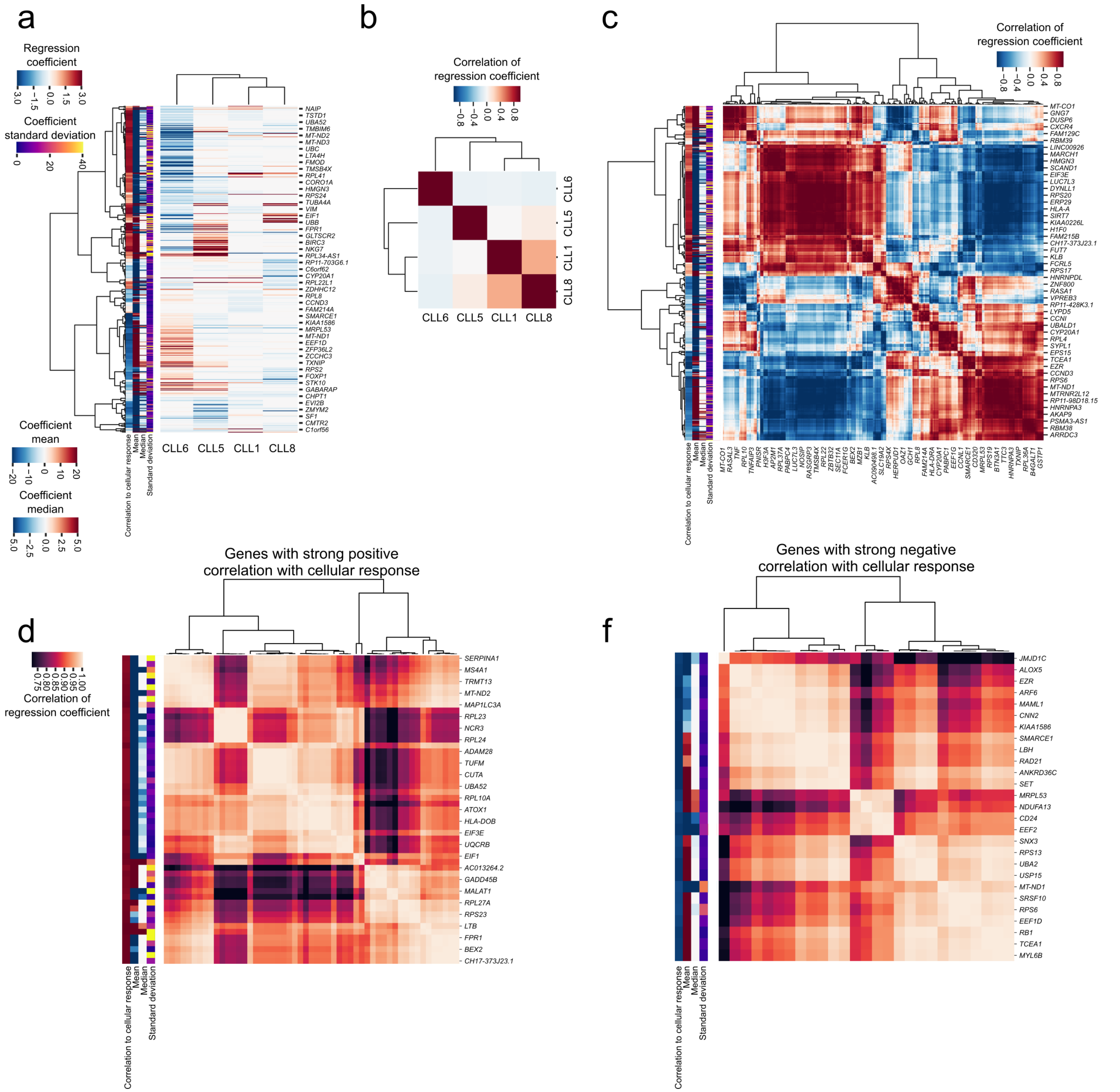

Supplementary Figure 13: Analysis of regression coefficients for gene expression upon ibrutinib treatment

a) Clustered heatmap of regression coefficients estimated for each patient and each gene over the course of the ibrutinib time series. For each gene, the heatmap shows a set of summary statistics calculated across patients (median, mean, standard deviation) as well as the Pearson correlation to the reduction of CLL cells at day 120 of ibrutinib treatment. **b)** Pairwise Pearson correlation of regression coefficients between patients, aggregated across all genes. **c)** Pairwise Pearson correlation of regression coefficients, showing genes separately (as in panel a). **d-e)** Pairwise Pearson correlation of regression coefficients (as in panel c), shown separately for genes that are highly correlated with slow (panel d) or fast (panel e) response to ibrutinib, defined by an absolute Pearson correlation above 0.9. Due to space constraints, an equidistant subset of genes is shown in the heatmaps of panel a, c, and d.
