# Supplementary material for: Chromatin mapping and single-cell immune profiling define the temporal dynamics of ibrutinib response in CLL
Source: Nat Commun. 2020 Jan 29;11:577. doi: 10.1038/s41467-019-14081-6 (PMC6989523; doi:10.1038/s41467-019-14081-6)
Supplement: Supplementary file 3 — Reporting Summary [file 41467_2019_14081_MOESM3_ESM.pdf]

nature research

Corresponding author(s): Christoph Bock

Last updated by author(s): Nov 28, 2019

Reporting Summary

Nature Research wishes to improve the reproducibility of the work that we publish. This form provides structure for consistency and transparency in reporting. For further information on Nature Research policies, see Authors & Referees and the Editorial Policy Checklist.

Statistics

For all statistical analyses, confirm that the following items are present in the figure legend, table legend, main text, or Methods section.

n/a	Confirmed	
☐	☒	The exact sample size (*n*) for each experimental group/condition, given as a discrete number and unit of measurement
☐	☒	A statement on whether measurements were taken from distinct samples or whether the same sample was measured repeatedly
☐	☒	The statistical test(s) used AND whether they are one- or two-sided<br>*Only common tests should be described solely by name; describe more complex techniques in the Methods section.*
☒	☐	A description of all covariates tested
☐	☒	A description of any assumptions or corrections, such as tests of normality and adjustment for multiple comparisons
☐	☒	A full description of the statistical parameters including central tendency (e.g. means) or other basic estimates (e.g. regression coefficient) AND variation (e.g. standard deviation) or associated estimates of uncertainty (e.g. confidence intervals)
☐	☒	For null hypothesis testing, the test statistic (e.g. *F*, *t*, *r*) with confidence intervals, effect sizes, degrees of freedom and *P* value noted<br>*Give P values as exact values whenever suitable.*
☒	☐	For Bayesian analysis, information on the choice of priors and Markov chain Monte Carlo settings
☒	☐	For hierarchical and complex designs, identification of the appropriate level for tests and full reporting of outcomes
☒	☐	Estimates of effect sizes (e.g. Cohen's *d*, Pearson's *r*), indicating how they were calculated

Our web collection on statistics for biologists contains articles on many of the points above.

Software and code

Policy information about availability of computer code

Data collection	Flow cytometry data was collected with the FlowJo (Tree Star) software.<br>Next generation sequencing data was collected with proprietary Illumina software.
Data analysis	The analysis source code is provided on the Supplementary Website (http://cll-timecourse.computational-epigenetics.org/) and on GitHub (https://github.com/epigen/cll-ibrutinib_time).

For manuscripts utilizing custom algorithms or software that are central to the research but not yet described in published literature, software must be made available to editors/reviewers. We strongly encourage code deposition in a community repository (e.g. GitHub). See the Nature Research guidelines for submitting code & software for further information.

Data

Policy information about availability of data

All manuscripts must include a data availability statement. This statement should provide the following information, where applicable:

- Accession codes, unique identifiers, or web links for publicly available datasets
- A list of figures that have associated raw data
- A description of any restrictions on data availability

All data are available through the Supplementary Website (http://cll-timecourse.computational-epigenetics.org/). Single-cell RNA-seq and ATAC-seq data have been deposited in the NCBI GEO database and are publicly available under accession number GSE111015 [https://www.ncbi.nlm.nih.gov/geo/query/acc.cgi?acc=GSE111015].

Field-specific reporting

Please select the one below that is the best fit for your research. If you are not sure, read the appropriate sections before making your selection.

☒ Life sciences ☐ Behavioural & social sciences ☐ Ecological, evolutionary & environmental sciences

For a reference copy of the document with all sections, see nature.com/documents/nr-reporting-summary-flat.pdf

Life sciences study design

All studies must disclose on these points even when the disclosure is negative.

Sample size	No sample-size calculation was performed.
Data exclusions	Data from one patient was excluded due to lower quality of the original material.
Replication	Each patient was profiled once for each timepoint of treatment. Findings were validated on published data from an independent cohort.
Randomization	Processing of samples from the individual patients did not follow any particular order, and batch effects were corrected as described in the Methods section.
Blinding	Because there were no pre-defined sample groups during data collection, blinding was not applicable for this study.

Reporting for specific materials, systems and methods

We require information from authors about some types of materials, experimental systems and methods used in many studies. Here, indicate whether each material, system or method listed is relevant to your study. If you are not sure if a list item applies to your research, read the appropriate section before selecting a response.

Materials & experimental systems

n/a	Involved in the study	
☐	☒	Antibodies
☒	☐	Eukaryotic cell lines
☒	☐	Palaeontology
☒	☐	Animals and other organisms
☐	☒	Human research participants
☒	☐	Clinical data

Methods

n/a	Involved in the study	
☒	☐	ChIP-seq
☐	☒	Flow cytometry
☒	☐	MRI-based neuroimaging

Antibodies

Antibodies used	Single-cell suspensions were stained with combinations of antibodies against CD3 (FITC, clone UCHT1, 1:200, Cat# 300452), CD4 (PE-TxRed, clone OKT4, 1:200, Cat# 317448), CD5 (PE-Cy7, clone UCHT2, 1:100, Cat# 300622), CD8 (APC-Cy7, clone SK1, 1:150, Cat# 344746), CD14 (PerCp-Cy5.5, clone M5E2, 1:100, Cat# 301824), CD19 (APC, clone HIB19, 1:100, Cat# 302212), CD25 (PE-Cy7, clone BC96, 1:100, Cat# 302612), CD38 (PE, clone HB-7, 1:100, Cat# 356604), CD45RA (PerCp-Cy5.5, clone HI100, 1:100, Cat# 304122), CD45RO (AF700, clone 304218, 1:100, Cat# 304218), CD56 (AF700, clone NCAM16.2, 1:100, Cat# 340363, BD Bioscience), CD127 (APC, clone A019D5, 1:100, Cat# 351342), CD197 (CCR7, PE, clone G043H7, 1:100, Cat# 353204), and DAPI viability dye (all obtained from Biolegend unless stated otherwise) for 30 min at 4 °C.
Validation	All antibodies are commercially available, and validation data are provided by the vendor.

Human research participants

Policy information about studies involving human research participants

Population characteristics	Relevant patient covariates in the study are age, sex, Binet stage, prior therapies, IGHV homology and mutation, cytogenetics profile, and TP53 mutations.
Recruitment	All patients were treated at the Department of Haematology and Stem Cell Transplantation, Central Hospital of Southern Pest, Budapest, Hungary, according to the revised guidelines of the International Workshop Chronic Lymphocytic Leukemia/National Cancer Institute.
Ethics oversight	The study was approved by the ethical committees of the contributing institutions (Dél-Pesti Centrumkórház, Semmelweis University, and Medical University of Vienna).

Note that full information on the approval of the study protocol must also be provided in the manuscript.

Flow Cytometry

Plots

Confirm that:

☒ The axis labels state the marker and fluorochrome used (e.g. CD4-FITC).

☒ The axis scales are clearly visible. Include numbers along axes only for bottom left plot of group (a 'group' is an analysis of identical markers).

☒ All plots are contour plots with outliers or pseudocolor plots.

☒ A numerical value for number of cells or percentage (with statistics) is provided.

Methodology

Sample preparation	Patient PBMCs were thawed and washed twice with PBS containing 0.1% BSA and 5 mM EDTA (PBS + BSA + EDTA). Cells were then incubated with anti-CD16/CD32 (clone 93, 1:200, Cat# 101301) to prevent nonspecific binding. Single-cell suspensions were stained with combinations of antibodies against CD3 (FITC, clone UCHT1, 1:200, Cat# 300452), CD4 (PE-TxRed, clone OKT4, 1:200, Cat# 317448), CD5 (PE-Cy7, clone UCHT2, 1:100, Cat# 300622), CD8 (APC-Cy7, clone SK1, 1:150, Cat# 344746), CD14 (PerCp-Cy5.5, clone M5E2, 1:100, Cat# 301824), CD19 (APC, clone HIB19, 1:100, Cat# 302212), CD25 (PE-Cy7, clone BC96, 1:100, Cat# 302612), CD38 (PE, clone HB-7, 1:100, Cat# 356604), CD45RA (PerCp-Cy5.5, clone HI100, 1:100, Cat# 304122), CD45RO (AF700, clone 304218, 1:100, Cat# 304218), CD56 (AF700, clone NCAM16.2, 1:100, Cat# 340363, BD Bioscience), CD127 (APC, clone A019D5, 1:100, Cat# 351342), CD197 (CCR7, PE, clone G043H7, 1:100, Cat# 353204), and DAPI viability dye (all obtained from Biolegend unless stated otherwise) for 30 min at 4 °C followed by two washes with PBS + BSA + EDTA.
Instrument	For flow cytometry, cells were acquired with an LSRFortessa Cell Analyzer (BD). For FACS, cells were sort-purified with a MoFlo Astrois (Beckman Coulter).
Software	Data analysis was performed with FlowJo (Tree Star) software.
Cell population abundance	CLL cells constituted the most abundant cell type in the majority of samples (Supplemental Figure 1b). It was not feasible to routinely perform post-purity checks on the sorted immune cell populations due to their low abundance.
Gating strategy	The gating strategy is depicted in Supplementary Figure 1a.

☒ Tick this box to confirm that a figure exemplifying the gating strategy is provided in the Supplementary Information.
