# Supplementary material for: Chromatin mapping and single-cell immune profiling define the temporal dynamics of ibrutinib response in CLL
Source: Nat Commun. 2020 Jan 29;11:577. doi: 10.1038/s41467-019-14081-6 (PMC6989523; doi:10.1038/s41467-019-14081-6)
Supplement: Supplementary file 4 — Description of Additional Supplementary Files [file 41467_2019_14081_MOESM4_ESM.docx]

**Description of Additional Supplementary Files**

**Supplementary Data 1:** Clinical annotation of the patients with CLL included in the time course analysis

**Supplementary Data 2:** Cell type composition over the time course as measured by flow cytometry

**Supplementary Data 3:** Expression of cell surface marker proteins as measured by flow cytometry

**Supplementary Data 4:** Cell type specific gene expression over the time course based on scRNA-seq

**Supplementary Data 5:** Cell type specific differential expression over the time course based on scRNA-seq

**Supplementary Data 6:** Summary statistics for ATAC-seq chromatin mapping in CLL cells

**Supplementary Data 7**: Dynamic chromatin regions over the time course in CLL cells

**Supplementary Data 8:** Summary statistics for ATAC-seq chromatin mapping in non-malignant immune cells

**Supplementary Data 9**: Dynamic chromatin regions over the time course in non-malignant immune cells
